# Critical comments on COViD-19 outbreak: succinct advice for dentists and oral healthcare professionals by Cirillo (2020)

**DOI:** 10.6026/97320630016509

**Published:** 2020-07-31

**Authors:** Vandan R Kasar, Olivia S Cajulis, Francesco Chiappelli

**Affiliations:** 1 UCSF School of Dentistry, San Francisco, CA; 2 Dental Group of Sherman Oaks, Sherman Oaks, California; 3 UCLA, Center for the Health Sciences, CSUN, Department of the Health Sciences

## Abstract

This article informs dental professionals of timely and critical recommendations to keep the practice of dentistry safe for the patients, the staff, the hygienists and the dentists. Teaching of these recommendations are being integrated in the clinical curriculum of pre-DDS and pre-DMD professionals, as well as in dental hygiene and dental assisting schools. The paper provides an essential and clearly-written overview of new mandatory procedures and protocols for the practice of dentistry, which will spread world-wide in the near future.

## Critical comments:

This paper [3] is an informative piece of writing on the necessary changes in the practice of dentistry during the present CoViD-19 pandemic. The paper expands and details recent CDC guidelines for Interim Infection Prevention and Control Guidance for Dental Settings during the CoViD-19 response with guidance and recommendations for providing non-emergency dental care to both patients with CoViD-19 and patients without CoViD-19. Guidance and recommendations also explore issues of patient management, administrative, practice re-structuring and workflow for optimal, work practice controls, sterilization of instruments, and personal protective equipment (PPE).

Dental practices, Cirillo argues, proffer several routes of possible transmission of the SARS-Cov2 virus, the causative agent for CoViD-19. Patients who enter the practice may be infectious vectors, and any surface they touch may become infectious fomites where the virus may survive for several hours. During the dental examination and procedure, these patients cause substantial risk of infection to the dentist, hygienists and dental assistants. Upon check-in and check-out, infectious patients, whether symptomatic or asymptomatic, cause significant risk of infection to the front-desk staff.

Airborne transmission, through respiratory droplets, sneezes, and most importantly, aerosolized saliva pose major exposure risks to oral healthcare providers, as also underscored by CDC and ADA (e.g., Guidance on preparing workplaces for CoViD-19, 2020).In addition, the virus can also cause indirect contamination through surface contact on dental equipment,counters, doors, and other surfaces. Therefore, it is imperative that oral health practitioners take important measures to protect themselves, their staff, and their patients to ultimately protect the public.

International, national and local bodies have create guidelines for dental care workers, which must be disseminated to limit the spread of the virus and to protect the health of those in our communities. Rigorous PPE policies are mandatory in the dental practice, as well as supplementary health screenings, such as acrylic barriers. Stringent infection control practices must be in place before any dental practice can re-open its door to providing dental care routinely. Rapid screening of CoViD-19 symptoms and referral for immediate testing for presence of SARS-Cov2 RNA in oral and laryngeal mucosa is both necessary and essential before resuming non-emergency dental care [1], because of the heightened infectious vulnerability risks among dental professionals [2].

Planning and preparing the environment of the dental practice (e.g., decontaminating vacuum and suction controls), updating staff protocols, and revising practice daily and hourly workflow, are essential to ensure strict safety precautions. In addition, patient triaging, checking the every patient’s symptom status before they enter the office, and limiting the number of patients in the practice at any one time is most timely and critical as pandemic progresses and until it is fully under control. Facemasks must be required of all patients who enter the practice, be removed only during the dental procedure. All staff must wear facemasks at all time, and N95 masks during the dental procedure.

Treatment rooms must be cleaned and disinfected after each patient, and the practice environment must be thoroughly disinfected at the end of the day. The US Environmental Protection Agency has prepared a list of Disinfectants for Use Against SARS-CoV2, which is given in its List N (https://www.epa.gov/pesticide-registration/list-n-disinfectants-use-against-sars-cov-2), and includes the specific formulation and contact use of hydrogen peroxide, quaternary ammonium, and sodium hypochlorite on frequently touched objects and surfaces.
